# Some of the Dangers of Bicycling

**Published:** 1897-06

**Authors:** 


### SOME OF THE DANGERS OF BICYCLING.

THE advantages to be derived from bicycling as a means of locomotion and pleasure are counterbalanced by the disadvantages arising from this sport. Aside from the accidents consequent to reckless and inexperienced riding, the health and happiness of many riders, especially ladies, are imperiled by an indiscriminate use of the popular wheel. The bicycle itself is as harmless as a watch, but when entrusted to ignorant or innocent riders becomes a dangerous instrument.

Perhaps the first great fault committed, or the first misstep, is for the individual to decide whether or not he or she is able physically, perhaps mentally, sometimes morally, to ride a wheel.

The fact that a certain friend rides is usually taken as the test and "if he or she is able to ride ten, twenty or thirty, or even fifty miles per day, certainly I can ride five miles." The next misstep is the obtaining of advice and opinion, and instead of consulting the family physician the bicycle dealer is chosen as the trusted adviser. In his desire to make a quick sale, because his season lasts only while the sun shines, he perhaps unconsciously suggests a wheel which is as much appropriate as the traveling optician's spectacles are to his unsuspecting purchasers.

Granted that there exists no organic disease of any of the vital organs, nevertheless the whole system may be in such a relaxed condition as to yield to any extra strain that may be put upon it. In this connection mention might be made to the cardiac accidents, where no valvular lesions are discoverable, due to want of tonicity in the cardiac muscles, resulting in dilatation, rupture or even valvular laceration. Another series of disturbances arise from the continuous strain or tension of the rider to keep the wheel erect.

The tension is not so very pronounced at any one time, but exists as long as the wheelman is astride his wheel, and the long continuance acts to disadvantage. To ride a wheel safely calls forth a double strain, a general one on the nerves and a particular one on the balancing center. The latter strain is as injurious as the former and a long train of nervous symptoms, neurasthenic in character, is the result. The "bicycle face" has been applied to the expression of riders who suffer from these nervous accidents.

Many bicyclers assert that they ride only moderately and yet have no idea what moderation means. To some it may be a two-mile spin, to others ten miles. Pedaling is so easy, the roads in such good condition, the summer scenery so enchanting, that the milestones pass by before fatigue sets in; when it does, the rider is generally at the distal end of the route and the return home is made with the system relaxed and fagged out or refreshed by means of alcoholic stimulants. It is a fallacy to make muscular effort the measure of excess and moderation in this form of exercise.

These are but a few of the dangers lurking in the bicycle even when the adjustment, saddle, handle-bars and gearing are fitted to each individual rider. That such is however not the case, may be judged from the following editorial extract from the L. A. W. Bulletin:

"No person should actually buy a bicycle until they can ride. When the beginner has mastered the rudiments of the art of keeping the wheel upright without assistance, the dealer of the future will have a device with pedals, handles and saddle, upon which the would-be purchaser will be asked to sit. The height of saddle will be adjusted while the rider is in position, as will also its fore and aft location; then the length of cranks will be adjustable while they are in motion. Various styles of saddles will be so fitted that they may be exchanged one for the other instantly while the rider stands on the pedals. A friction scale arrangement can be fixed so as to give to the pedals the same resistance they would meet on grades of varying steepness.

With a machine of this kind the most comfortable saddle, the proper location of it, the proper length of crank, and the most suitable gear for any given rider can be determined in a few minutes, so that when a wheel is delivered the customer will get the most out of cycling, and hence be a better advocate in the sight of others, and the dealer and the manufacturer will have less complaints."

---

**Woman's Place in Medicine.**—Remarks of Dr. Maud J. Frye at the banquet of the Alumni Association of Buffalo University Medical College, April 27, 1897, to the sentiment "Me judice":

I wish to present to you tonight some thoughts concerning woman's place in medicine ; the limit of her work, the line which she will oftenest follow.

Whether we like to confess it or not, woman's physical organisation necessarily sets a limit to her achievements. The women of Colorado who propose to serve in the National Guard of that progressive state, will probably remain to the end as they have been from the beginning, a home guard. In reckoning the chances of woman's success when she comes into competition with man, the physical factor has always to be taken into account. That medical work which makes the least demand on woman's physique is, other things being equal, that in which she will best succeed.

It is, however, concerning a feature of woman's mental equipment for work in medicine that I wish especially to speak. I do not expect many of you to agree with me, yet I ask you to think on these things. We must acknowledge between the average man and the average woman an unlikeness of mental capacity comparable to their physical unlikeness, a sex in mind, if I may use the term. This is not equivalent to saying that man is superior to woman. There is equality with diversity. The elm is not better than the oak, nor the oak than the elm, but each is good of its kind. There are mental processes which are identical in the sexes, there are others which may oftentimes give advantage to the woman in the work of the physician. Does the woman's way of seeing and doing ever handicap her ? Let us try to answer the question.

There is one quality which more than any other makes for success in certain lines of medical work. It is the type of courage more or less common to all men, rarely found in women—the courage which dares. It is the courage of the soldier, and the explorer. It is the courage which makes possible the great surgeon.

On the other hand, through all time woman's genius has given this character to her work, that it has not turned to great deeds but has contented itself with the humbler, often holier, tasks of the home, the school-room and the sick chamber. Her courage makes possible trained nursing, and in the Christian world is the right arm of the church. It is the courage whose other name is patient endurance.

In the early days when the woman who began the study of medicine was of heroic type, her success was assured from the beginning, for the courage which dared to commence the struggle was equal to any hardship or any emergency which arose later. She has opened the way for the woman of average ability, the woman who but for her pioneer work would be teacher or nurse. The average woman has not, as a rule, the daring of an Elizabeth Blackwell. The test of fitness for medical work which college and early professional life gave the one is denied the other, and the open door to medicine is not altogether gain.

The lack of daring does not make a physician less trustworthy, nay rather she may be more worthy of trust, but the lack does not work to her material advantage. There are departments of medicine, notably bacteriology, for which not only woman's type of courage, but her whole manner of training, from her youth up, should eminently fit her and in such work as this I believe she will excel. But the woman who gains wealth and fame in the practice of general medicine or of surgery will be the woman who possesses, without necessarily losing anything of her outward womanliness, the masculine type of courage. She will be the woman who would rather be wrong than not try. The average woman would rather not try than risk being wrong.

There is a special message which I wish to give to the young women receiving their diplomas tonight. It is concerning your work among women. Whether you intend to be a gynecologist or not, every woman who enters your office expects you to be. Setting aside your right to do whatever you can and desire to do, the fact that women wish women physicians is the only reason for your being such. You will come to feel later the great power for good—not altogether in a professional way, but as friend and counselor—which is yours, for the good woman who is a good physician has before her, in teaching to her sex right ways of living and true views of life, a work which will bring satisfaction to herself and blessing to others.

To the end of time there is to be marrying and giving in marriage, and except for the woman of unusual talent wifehood and motherhood are of themselves a vocation. Because of this and because other occupations demanding less of her physically and mentally and offering quicker success are open to woman, the number of women physicians will always be few. Yet whether we ever occupy a great place in medicine or not, a place is ours. We are better women for the knowledge of humanity which our work has given us. May we not hope that humankind will be the better, too, for our having wrought!

> "All service is the same with God—
> To God, whose puppets, best and worst,
> We are; there is no last nor first."

